# Preharvest Elicitors as a Tool to Enhance Bioactive Compounds and Quality of Both Peel and Pulp of Yellow Pitahaya (*Selenicereus megalanthus* Haw.) at Harvest and during Postharvest Storage

**DOI:** 10.3390/ijms25105435

**Published:** 2024-05-16

**Authors:** Alex Erazo-Lara, María Emma García-Pastor, Pedro Antonio Padilla-González, Daniel Valero, María Serrano

**Affiliations:** 1 Escuela Politécnica Superior de Chimborazo (ESPOCH), Sede Morona Santiago, Macas 140101, Ecuador; alex.erazol@espoch.edu.ec; 2 Department of Food Technology, Escuela Politécnica Superiorde Orihuel—Centro de Investigación e Innovación Agroalimentario y Agroambiental, University Miguel Hernández, Ctra. Beniel Km. 3.2, 03312 Orihuela, Spain; ppadilla@umh.es; 3 Department of Applied Biology, Escuela Politécnica Superiorde Orihuel—Centro de Investigación e Innovación Agroalimentario y Agroambiental, University Miguel Hernández, Ctra. Beniel Km. 3.2, 03312 Orihuela, Spain; m.garciap@umh.es

**Keywords:** methyl salicylate, salicylic acid, methyl jasmonate, oxalic acid, bioactive compounds, phenolics, carotenoids

## Abstract

Yellow pitahaya is a tropical fruit that has gained popularity in recent years. Natural elicitors are compounds that can stimulate the resistance and quality of fruits. The objective of this study was to evaluate the effects of natural elicitors, methyl salicylate (MeSa), methyl jasmonate (JaMe), salicylic acid (SA) and oxalic acid (OA) at concentrations of 0.1 mM (MeSa and JaMe) and 5 mM (SA and OA), applied to the yellow pitahaya fruits under greenhouse conditions. After full blossom, four applications were made with a frequency of 15 days. At the time of harvest and after storage, the following variables were evaluated: firmness (whole fruit), total soluble solids (TSS), total acidity (TA), phenolics and carotenoids (in the pulp), while phenolics, carotenoids, macronutrients and micronutrients were determined in the peel. The results showed MeSa advanced the fruit maturation, according to higher TSS, lower TA and firmness than MeJa-treated fruits, for which a delayed ripening process was shown. All treatments induced a higher polyphenolic concentration during storage. Regarding the alternative use of the peel as a by-product, the application of natural elicitors significantly increased the content of polyphenols, carotenoids, macronutrients and micronutrients in the peel, especially MeSa, which can be used as a bioactive compound in the food industry. In conclusion, the results indicate that natural elicitors can be an alternative to improve the quality and shelf life of yellow pitahaya fruits.

## 1. Introduction

Pitahaya (*Selenicereus megalanthus*) is a native plant of the *Cactaceae* family, known worldwide as “dragon fruit” [1]. The province of Morona Santiago, especially the Palora canton, is among the main pitahaya-producing areas in Ecuador, this activity being an important source of employment and economic income for the country [2]. Yellow dragon fruit has gained popularity in the food industry and export due to its water content, nutrients and bioactive compounds, such as glucose, vitamins, organic acids, soluble dietary fibre and constituent minerals [3]. Pitahaya is considered an exotic fruit that originates from Central America and part of South America. It was initially found in the wild by Spanish conquistadors, who christened the term “pitahaya”, which refers to its scaly appearance. Currently, the existence of more than one species of pitahaya is recognized, given that human intervention and influence have given rise to its diversity that encompasses both morphological and organoleptic aspects: red pitahaya (*Hylocereus undatus*) and the yellow. Compared with the red pitahaya, the Palora ecotype has higher values of firmness, total acidity (TA), total soluble solids (TSS), vitamin C, antioxidant capacity and total polyphenols [4]. Yellow pitahaya is characterized by its attractive colour and the composition of bioactive moieties, such as flavonoids and other polyphenolic compounds [5]. When consumed, it can provide functional actions, supporting immunity and performing antimicrobial, antioxidant, hepatoprotective, hypoglycaemic, healing and antiproliferative activities [6].

Although the cultivation and commercialization of the yellow pitahaya has gained relevance in the agri-food industry, due to the growing demand in both the national and international markets, there are significant challenges in relation to the quality and management of this exotic fruit, especially with regard to optimizing its production and sustainable use, the growth and ripening process and postharvest handling [7]. In this sense, the use of natural elicitor compounds could be a good alternative. Elicitors present a viable option to promote sustainable agriculture to replace the use of agrochemicals in the production of food and other resources useful to people. To date, there has been no report indicating that the use of elicitors, regardless of whether they are of biotic or abiotic origin, generates adverse effects on plants, human health or the surrounding environment [8].

Agricultural crops are very sensitive to different abiotic stresses due to abnormal climatic changes, the increase in atmospheric temperature being one of the most crucial factors, affecting crop yield performance, food quality, security, availability and nutrient deficiencies, among others [9,10]. Therefore, it is necessary to mitigate climate change and adopt efficient measurements. Although there are many research limitations, the use of natural elicitors has been proved to be effective at counteracting the negative effects of climate change. These elicitors include methyl salicylate (MeSa), methyl jasmonate (JaMe), oxalic acid (OA) and salicylic acid (SA). The preharvest application of these compounds was effective on increasing crop yield and quality at the time of harvest and after storage in several fruit commodities, such as table grape, pomegranate and sweet cherry [11,12,13,14,15]. These elicitors can stimulate the defence responses of plants by the activation of phenolic compounds and antioxidant enzymes, which have the ability to counteract the negative effects of reactive oxygen species (ROS) [16].

As yellow pitahaya production advances, its relevance for industrial uses increases. This process involves the extraction of the pulp and generating a vast amount of disposable by-product, including the epicarp and part of the mesopcarp [17,18]. The pitahaya peel, which accounts 35–45% of the total fruit, contains fibre, polyphenols, macro- and micronutrients, which represent a potential source for various industries, such as pharmaceuticals, cosmetics and nutraceuticals, among others [19]. Pitahaya peels contain 75.2% fibre and high amounts of vitamin C and magnesium, which makes them suitable for the production of products rich in fibre, such as cookies and fritters [20].

Given the above, there is little scientific evidence on the effect of the application of natural elicitors on the quality of yellow pitahaya fruit and the use of the peel as a source of bioactive compounds for composting. This research evaluated the effect of four natural elicitors, methyl salicylate (MeSa), methyl jasmonate (JaMe), oxalic acid (OA) and salicylic acid (SA), on the quality of pitahaya at the time of harvest and after storage, as well as the peel’s bioactive compounds. By addressing these aspects, the aim is to contribute to the development of more sustainable agricultural practices and the improvement of the dragon fruit value chain, benefiting both producers and consumers and the environment.

## 2. Results

The objective of this study was to evaluate the effects of the natural elicitors methyl salicylate (MeSa), methyl jasmonate (JaMe), salicylic acid (AS) and oxalic acid (AO) at concentrations of 0.1 mM and 5 mM, respectively, applied four times during the growth of yellow pitahaya fruits under greenhouse conditions located in Palora, Ecuador. The efficacy of these treatments on pitahaya quality at harvest was evaluated, as well as the peel to be used as a by-product. Finally, the pitahaya fruits were stored to evaluate the fruit quality and the expected shelf life. 

### 2.1. Fruit Quality at Harvest and during Postharvest Storage

**Figure 1:**
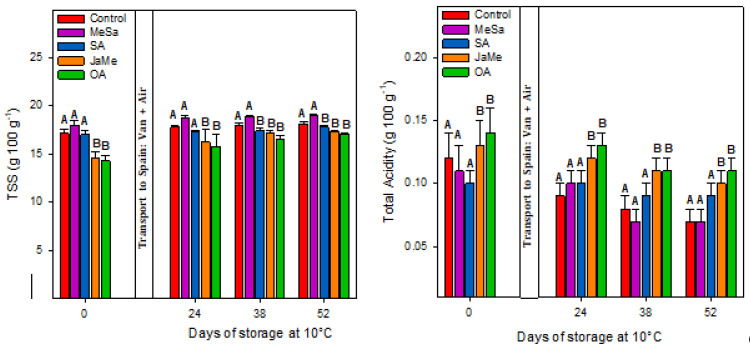
Total soluble solids (TSS) and total acidity (TA) concentration in pitahaya fruits as affected by preharvest treatments with methyl salicylate (MeSa), salicylic acid (SA), methyl jasmonate (JaMe) and oxalic acid (OA) and the control (untreated). For each sampling date, bars (mean ± SE) with different letters show significant differences at *p* ≤ 0.05.

**Figure 2:**
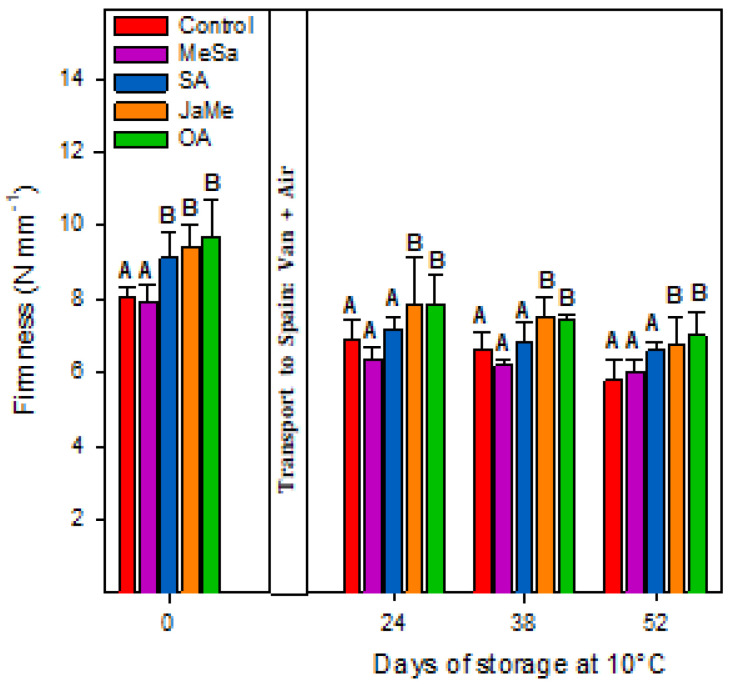
Firmness (N mm^−1^) values in pitahaya fruits as affected by preharvest treatments with methyl salicylate (MeSa), salicylic acid (SA), methyl jasmonate (JaMe) and oxalic acid (OA) and the control (untreated). For each sampling date, bars (mean ± SE) with different letters show significant differences at *p* ≤ 0.05.

The pitahaya quality at harvest and during storage at 10 °C was evaluated by three parameters: TSS and TA (Figure 1) and fruit firmness (Figure 2).

At harvest time, the fruits treated with MeSa and SA and the control showed significantly higher TSS (17–18 °Brix) and lower TA (≈0.10 g 100 g^−1^) compared with those treated with JaMe and OA, in which lower TSS (14–15 °Brix) and higher TA (≈0.14 g 100 g^−1^) were obtained. Similarly, firmness (Figure 2) was higher in the pitahayas treated with SA, JaMe and OA (≈9–19 N mm^−1^), while the contrary occurred with the fruit treated with MeSa and the control (8 N mm^−1^). During postharvest storage, the same behaviour was observed, with MeSa and the control being the treatments that exhibited higher TSS and lower TAA and firmness compared with the pitahayas treated with SA, JaMe and OA. It was noticeable that for all treatments, TSS slightly increased during storage, while for TA and firmness, a significant diminution was obtained.

### 2.2. Mineral Composition and Bioactive Content of the Peel

**Figure 3:**
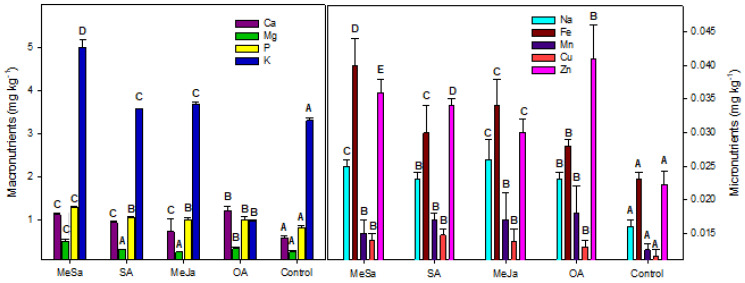
Concentration of Ca, Mg, P and K (macronutrients) and Na, Fe, Mn, Cu and Zn (micronutrients) in the peel of pitahaya fruits as affected by preharvest treatments with methyl salicylate (MeSa), salicylic acid (SA), methyl jasmonate (JaMe) and oxalic acid (OA) and the control (untreated). For each mineral, bars (mean ± SE) with different letters show significant differences among treatments at *p* ≤ 0.05.

**Figure 4:**
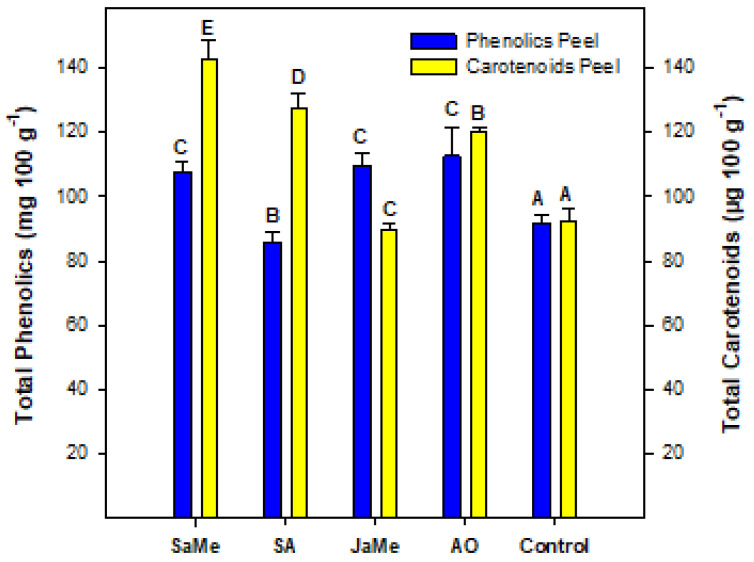
Concentration of total phenolics and carotenoids in the peel of pitahaya fruits as affected by preharvest treatments with methyl salicylate (MeSa), salicylic acid (SA), methyl jasmonate (JaMe) and oxalic acid (OA) and the control (untreated). For each bioactive compound, bars (mean ± SE) with different letters show significant differences among treatments at *p* ≤ 0.05.

The peel of the recently harvested pitahayas was used for the determination of the mineral composition (Figure 3) and total phenolics and carotenoids (Figure 4).

The content of all macronutrients Ca, P and K was significantly higher in the peel of all pitahaya treated fruits than in the control samples, while Mg was significantly higher in the MeSa- and OA-treated peels (Figure 3). The cation K was found to be the major mineral followed by P, with the content of Mg being a minor macronutrient. Among the treatments, MeSa was the most effective elicitor in increasing the content of all micronutrients.

With respect to the bioactive compounds, all treatments were effective in increasing the concentration of total carotenoids in the peel of the pitahaya fruits, with the exception of JaMe (Figure 4). On the other hand, the same behaviour was observed for the concentration of total phenolics, with the exception of SA-treated peels, which showed the lowest content.

### 2.3. Bioactive Compounds and Antioxidant Activity at Harvest and during Postharvest Storage

**Figure 5:**
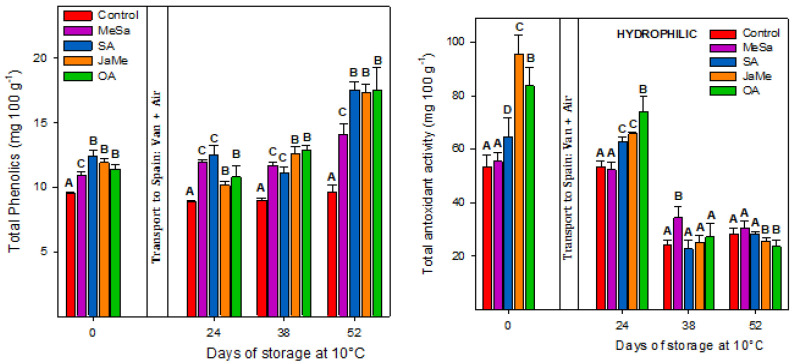
Total phenolics and hydrophilic antioxidant activity concentration in the pulp of pitahaya fruits as affected by preharvest treatments with methyl salicylate (MeSa), salicylic acid (SA), methyl jasmonate (JaMe) and oxalic acid (OA) and the control (untreated). For each sampling date, bars (mean ± SE) with different letters show significant differences at *p* ≤ 0.05.

At harvest, the content of total phenolics and total antioxidant activity (hydrophilic) was higher in the SA-, JaMe- and OA-treated pitahayas than those obtained in the MeSa-treated and control fruits (Figure 5). During storage, the phenolic concentration for the control fruits remained unchanged, while the total antioxidant activity showed a significant diminution, reaching the lowest activity (≈30 mg of Trolox eq. 100 g^−1^). On the contrary, the total polyphenol content (Figure 5) increased during storage for all treated pitahaya fruits (≈18 mg of gallic acid eq. 100 g^−1^). The determination of the total antioxidant activity revealed that all treated pitahayas saw significant decreases in their values, as did the control fruits, and also reached the minimum activity at the end of storage (≈30 mg of Trolox eq. 100 g^−1^).

**Figure 6:**
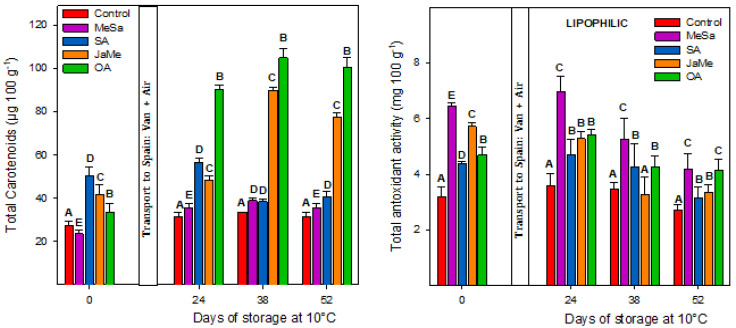
Total carotenoids and lipophilic antioxidant activity concentration in the pulp of pitahaya fruits affected by preharvest treatments with methyl salicylate (MeSa), salicylic acid (SA), methyl jasmonate (JaMe) and oxalic acid (OA) and the control (untreated). For each sampling date, bars (mean ± SE) with different letters show significant differences at *p* ≤ 0.05.

At harvest, the content of total carotenoids in the pulp (Figure 6) was significantly higher in the SA-, JaMe- and OA-treated pitahayas (≈40–60 μg β-carotene eq. 100 g^−1^) than those obtained in the MeSa-treated and control fruits (≈20 μg β-carotene eq. 100 g^−1^). The highest carotenoid content was shown for pitahayas treated with SA. With respect to the total antioxidant activity (lipophilic) at harvest, all pitahayas treated showed significantly higher activity (≈5–7 mg of Trolox eq. 100 g^−1^) than the control fruits (≈3 mg of Trolox eq. 100 g^−1^).

During storage, the carotenoid concentration for the control fruits remained unchanged, while treated pitahayas showed a significant increase, especially with the application of OA (≈90–100 μg β-carotene eq. 100 g^−1^) and JaMe (≈90 μg β-carotene eq. 100 g^−1^). The total antioxidant activity (lipophilic) during storage revealed that for all control and pitahaya treated fruits, a significant reduction was shown, although the values were always significantly higher in the treated than in the control fruits. Interestingly, the elicitor that induced the highest lipophilic activity was MeSa.

Finally, the hydrophilic antioxidant activity was higher (≈50-fold) than the lipophilic one in the pulp of yellow pitahayas. The comparison between peel and pulp demonstrated that the concentration of total phenolics and carotenoids was 10-fold and 2.5-fold higher, respectively, in the peel than in the pulp.

## 3. Discussion

The pitahaya is divided in two genera: *Hylocereus* (red skin and white or pink flesh) and *Selenicereus* (yellow skin and white flesh). Most of the research has been carried out on red varieties [3,17] compared to the limited knowledge about the growth process, maturation, postharvest potential and shelf life of other varieties [4].

With the aim of providing new knowledge on the quality of yellow pitahaya at the time of harvest and its behaviour during postharvest storage, in this study, we consider the use of different elicitors, applied at preharvest, and their influence on several organoleptic, nutritional and functional quality parameters. In addition, some properties of the peel of yellow pitahaya were evaluated with the objective to be used as a by-product.

At harvest time, the preharvest application of the elicitors affected differentially the quality traits in yellow pitahaya, since JaMe and OA showed higher values of firmness and TA and lower TSS, while MeSA and SA showed the contrary and exhibited similar behaviour to the control fruits. These results demonstrate that the on-tree ripening process was delayed in the fruits treated with JaMe and OA, while MeSa and SA advanced the ripening process of pitahaya. The ripening process is accompanied by fruit softening and is related to the degradation of cell wall components, such as pectin and cellulose, by the action of the cell wall enzymes polygalacturonase (PG), pectin methylesterase (PME) and cellulase (CEL) causing the decomposition of cellulose and hemicelluloses [21]. This softening process is accompanied by the increase in TSS parallel to the TA diminution. The differences found at harvest were also maintained during postharvest storage, in which after 52 days, the pitahayas treated with JaMe and OA maintained a higher firmness and TA and lower TSS than MeSa and SA.

SA and its derivative MeSa are naturally occurring and considered safe [22] and, when applied as preharvest treatment, could have roles as plant growth regulators and as inducers of SAR, in turn alleviating the devastating effects of abiotic stresses [16,23]. During pitahaya growth, ripening and postharvest storage, both SA and MeSA enhanced TSS but induced lower TA and firmness, these effects being related to an advancement of the ripening process. TSS and TA are good indicators of sweetness and sourness, respectively, with the most important parameter of fruit being taste, which determines consumer acceptability and purchase decisions [21]. During storage, the decrease in TSS and TA is mainly caused by the increase in respiration rate by utilizing the reserved substances [24]. SA and MeSa have been reported to modulate these quality traits, although the effects depended on fruit species, the type of elicitor, concentration and the number of applications. For instance, preharvest SA (1, 2 and 3 mM) applied to lime resulted in hastening maturity for all doses [25], similarly to grapes at 1, 1.5 and mM [26], and in contrast, SA at 1 mM accelerated ripening, but at 2 mM, a delay was observed in peach [27]. Table grapes treated with SA (0.01 mM) and MeSa (0.1 mM) increased TSS, while ASA (acetylsalicylic acid 1 mM), which is an SA derivative, decreased TSS at harvest [28].

In our previous report on pitahaya [29], preharvest SA and MeSa applied at 1, 5 and 10 mM increased TSS with the doses of 1 and 10 mM of MeSA and SA at 1 and 5 mM. In addition, the elicitors increased yield productivity and fruit weight. All elicitors induced benefits on pitahaya crop production, but the effect depended on the concentration tested. All elicitors increased pitahaya size in both polar and equatorial diameters, and the fruit weight with the highest fruit mass was found with oxalic acid at 5 mM. These results justify the concentration used in this study for storage. It seems that SA could increase the translocation of sugars from the leaves to the pitahaya and thus lead to the enhancement of TSS. During fruit growth and development, sucrose is accumulated due to the increase in the enzymes sucrose phosphate synthase and sucrose phosphatase [30], but during ripening, sucrose levels decrease parallelly to the increased levels of nonreducing sugar content, mainly glucose and fructose. However, the higher sugar translocation could be due also to the possible role of the elicitors in increasing vegetative (foliar) growth.

Methyl jasmonate (MeJA) is a volatile hormone derived from jasmonic acid involved in a wide range of plant functions, acting as a signal in response to abiotic stresses and modulating the biosynthesis of other plant growth regulators [31,32]. Plants synthesize this hormone in defence against biotic and abiotic stresses but also modulate fruit growth and ripening [33]. The higher TA and firmness and the lower TSS in MeJA-treated pitahaya may be attributed to the ripening–retarding effects of MeJA and a delay in the senescence process. It is noteworthy that MeJa positively affects fruit growth and crop quality, this issue being extensively reported in several fruit commodities. Also, the MeJa effects on these quality traits in pitahaya applied at preharvest remained also during postharvest storage. Accordingly, postharvest MeJA treatments have been demonstrated to modify the properties of fruits during postharvest storage [34].

In this sense, MeJA increased the sugar content in peach, leading to an enhancement of nutritional quality [35]. MeJa applied to Kinnow mandarin as preharvest treatment at 0.1, 0.3, 0.5 and 0.7 mM showed higher fruit firmness and TA and a lower TSS/TA ratio (indicative of the ripening index), 0.5 mM being the most effective in delaying the ripening [36]. Also, preharvest MeJA treatments resulted in higher fruit firmness values at harvest for all the evaluated cultivars, including ‘Early Lory’, ‘Prime Giant’ and ‘Sweetheart’, during four (2019–2022) different growing seasons [37]. It seems that MeJa leads to firmer fruit by direct action since this volatile compound can enhance the integrity of the cell wall [38]. Moreover, indirect action has been also proposed by which MeJa delayed fruit softening due to an elevated and stable level of Ca^2+^ content in the cell walls. Other authors suggest that MeJa activates pectin methylesterase (PME) enzymes, with the liberation of methyl esters from the pectins and generation of free pectins that could cross-link with Ca^2+^, thus increasing cell wall firmness [39].

On the other hand, oxalic acid (OA) is a naturally occurring organic acid belonging to the Krebs cycle, with multiple functions that alter plant metabolism. According to research carried out during the last two decades, OA has been proven to have antioxidant activity, focusing essentially on the enhancement of crop yield and quality, but it has also been shown to delay postharvest ripening and senescence [40,41]. In yellow pitahaya, the preharvest application of OA showed similar results to those obtained for MeJa-treated fruits, that is, a delay of the ripening based on lower TSS and higher TA and firmness.

The role of organic acids in general, and particularly OA, play an essential role in modulating fruit ripening and delaying senescence during postharvest storage, as well as upregulating the resistance against both abiotic and biotic stresses. Pre- and postharvest OA applications have been widely used with the objective to improve fruit quality at harvest. Preharvest OA in sweet cherry [14] and pomegranate [13] showed a clear delay in ripening and senescence, with net benefits in terms of quality. The higher firmness in OA-treated pitahayas probably related to the biosynthesis of oxalate-soluble pectin and the inhibition of pectin solubilization, with this maintaining higher fruit firmness [21]. This effect was shown in other fruits such as peach and lemon [42,43]. Higher levels of TA could be related to lower respiration rates, since organic acids are the primary substrates to be used in the respiration physiological process.

The edible part of the pitahaya is the pulp, which is mainly consumed as fresh fruit, juices, jams, ice cream and dessert [44]. However, the fruit has an important part, peel (which account for between 40 and 50% of the total mass), which currently is considered waste but could have some potential to be used as a by-product. In the case of yellow pitahaya, there is almost no literature about the composition of the peel, although there is knowledge in the case of red varieties [45].

According to the latest figures, about 1.3 billion tonnes of food and food by-products are wasted each year, the agroindustry sector being very significative of this, since fruit peels are a good source of high-value-added functional compounds to be used in food, pharmaceutical or cosmetic industries [46]. The fruit of yellow pitahaya is formed by the peel or skin, which accounts for 35–45% of the total mass, pulp that has a mass of 50–55% and black seeds. Dragon fruit peels are by-products of juice production that are usually wasted, but they are rich in polyphenols, vitamins and dietary fibres [47]. Several studies have shown that pitahaya can alleviate some diseases including cardiovascular diseases and metabolic syndromes due to the occurrence of bioactive compounds such as polyphenols, betacyanins or vitamins that can be found in both in the peel or pulp of red pitahayas species [48]. However, there is no available literature about the use of the peel of yellow pitahaya, which could have the potential to be used in the food industry as functional ingredients, nutraceutical compounds or edible films. Thus, the peels may be used as fat substitutes, enhancing the nutritional value and functional properties of food.

As a first approximation, we analysed the mineral composition, total phenolics and total carotenoids in the peel of yellow pitahaya cv. Palora. With respect to mineral composition in the peel of pitahaya, the preharvest elicitors contained a higher concentration of the macronutrients Ca, P and K, with Mg being significantly higher in the peel of MeSa- and OA-treated pitahayas. All micronutrients (Fe, Mn, Cu, Zn and Na) were higher in the treated pitahaya, with MeSa being the most effective elicitor in increasing the content of all minerals. Numerous studies found that pitahaya (red species) has more mineral content (K, P, Na, Mg, Fe and Ca) than other tropical fruits, such as pineapple or mango [49]. Shah et al., 2020 [50], reported that the total ash and mineral contents of the peel were 2-fold higher than those obtained in the pulp, studying several pitahayas species, either red or yellow fruits.

All treatments induced increased concentrations of total carotenoids in the peel of the pitahaya fruits, with MeSa being the most effective elicitor. There is no literature reporting the carotenoids in the peel of yellow pitahaya for comparative purposes, although there is some evidence in other red pitahayas. The total carotenoids content in the peel of *H. costaricensis* (red pitahaya) at commercial ripening were ≈2 mg 100 g^−1^ [51]. In a comparative study with three pitahaya peels (*Hylocereus undatus*, *Hylocereus costariscensis* and *Hylocereus megalanthus*), the total carotenoids were found in the range of 18–24 μg 100 g^−1^, with the main compounds being xantophyll and β-Carotene [52]. The market of natural pigments has been growing in the last decade as consumer demand alternatives to synthetic colorants, which are considered harmful. Then, exploring natural and eco-friendly pigments is therefore necessary [53]. Contrary to red pitahaya, which is rich in red pigments (betalains and anthocyanins), the peel of yellow pitahaya (rich in carotenoids) has not been studied in depth and should be considered as a potential source of carotenoids.

The preharvest application of MeSa, JaMe and OA showed a higher content of total phenolics in the peel, while SA-treated peels had lower concentrations. On average, total polyphenol concentrations ranged between 90 and 100 mg gallic acid eq. 100 g^−1^ in the peel of yellow pitahaya. It was reported [54] that total phenol and flavonoid (6 and 20 mg g^−1^, respectively) contents reached their maximum at stage 1 (immature fruits) and decreased progressively as fruit development advanced, reaching the lowest levels at harvest time.

The main phenolic compounds found in the peel of red pitahaya belongs to the flavonoid group. Recent studies identified 16 phenolic acids including derivatives of benzoic and ellagic acid [5,55]. The peel of three cultivars of *Hylocereus undatu* increased gradually the content of total phenolics during fruit ripening until reaching the maximum at the time of harvest, the concentration being higher than those obtained in the pulp, suggesting that pitaya peels could be considered as good sources of natural phenols [56]. The phenolic content in the peel of red (*Hylocereus monacanthus*) and yellow (*Hylocereus megalanthus*) pitahaya ecotypes confirmed that the peel had higher total polyphenols (2-fold) than the pulp of the red species, but the contrary occurred in the yellow pitahaya since the pulp had a 12% higher content than the peel [57]. The higher content of total phenolics in the peel of yellow pitahaya treated with the elicitors supports the idea that the peel could have a potential in the food industry to be used in nutritional supplements. Recently, the mucilage of the peel of yellow pitahaya has been postulated as an innovative hydrocolloid to be used in the food industry because it is a good source of dietary fibre with a potent antioxidant activity, as well as good solubility, a high water retention efficiency and an excellent capacity to form emulsions [58]. Also, biscuits made with 50% refined wheat flour and 50% peel powder from pitahaya increased 5-fold the fibre content as well as the amount of gallic acid, and the biscuit has been considered palatable and of a good quality [59].

In recently harvested fruits, the concentration of total phenolics and total antioxidant activity (hydrophilic) was higher in the pulp treated with SA, JaMe and OA and lower in those treated with MeSa and the control fruits. These differences were maintained for the entire period of postharvest storage (52 days at 10 °C), although the content of total phenolics increased during storage, while the contrary occurred for the total antioxidant activity. Interestingly, the levels of total phenolics in control pitahayas remained unchanged during storage, while MeSa-treated fruits enhanced the total phenolics. Accordingly, the application of MeSa during fruit growth has been shown to increase the content of polyphenols of several fruits at harvest and during cold storage, such as grape and sweet cherry, among others [11,16,28]. Similarly, JaMe and OA enhanced the total phenolics of table grapes, pomegranate and sweet cherry at the time of harvest and also during postharvest storage [12,13].

MeJa applied as postharvest treatment in red pitahaya induced higher total flavonoids, phenolics, anthocyanins and antioxidant activity measured by FRAP and DDPH assays [60]. In a comparison of three species (*H. costaricensis*, *H. undatus* and *H. megalanthus*), the content of total phenolics in the pulp was 33, 23 and 22 mg gallic acid eq. 100 g^−1^, *H. costaricensis* being the fruit with the highest antioxidant activity, 15-fold higher than *H. undatus* and *H. megalanthus* [61]. The three species differ in the colour of the peel and flesh, and it can be concluded that total phenol content and antioxidant capacity are notably higher in red-fleshed fruits than white-fleshed fruits. This was confirmed by [62], regarding their results for *Hylocereus polyrhizus Hylocereus undatus*, in which the main phenolic compound in both pitahayas was quercetin.

At the time of harvest, the total carotenoid content was affected by preharvest treatment, with SA, JaMe and OA being the elicitors showing higher concentrations than those treated with MeSa and the control fruits. The concentration of carotenoids in pitahaya species, either red or yellow, has not been investigated in depth, although some evidence exists. Four xantophylls (lutein, neoxanthin, violaxanthin and dihydroxy dihydrozeaxanthin) and two carotenes (lycopene, β-carotene) have been identified in both the peel and pulp of red pitahayas [63]. The pulp of three species (*H. undatus*, *H. costariscensis* and *H. megalanthus*) revealed a range of 32–60 μg 100 g^−1^ of β-carotene and 18.24 μg 100 g^−1^ of xantophyll [52]. In Indian species of dragon fruits (*H. costariscensis* and *H. megalanthus*), β-carotene has been found to be a major carotenoid. In white flesh dragon fruits from the Vietnam variety (white flesh), β-carotene, lycopene and vitamin E at concentrations of 1.4, 3.4 and 0.26 μg 100 g^−1^, respectively, were reported [64].

During storage, the carotenoids increased in OA- and JaMe-treated pitahayas, while the lipophilic antioxidant activity decreased during storage, although all treated fruits had higher concentrations than the control ones, the MeSa treatment being the elicitor that induced the highest amount of carotenoids. The continuous increase in carotenoids in the postharvest storage of yellow pitahaya maybe is due to the normal ripening process, in which the acceleration of chlorophyll degradation leads to increases in carotenoids.

Generally, there is a close relationship between carotenoids and lipophilic antioxidant activity, given the lipophilic nature of the carotenoids [21]. The increase in β-carotene and lycopene has been associated to an enhancement of the capacity to scavenge the ROS species that can be generated during the postharvest storage of fruits [65]. There is no literature about the effect of the elicitors on carotenoid content in red or yellow pitahaya, although our results confirm that JaMe applied at preharvest or postharvest increased the concentration of the total carotenoids of mandarin [36]. On the other hand, preharvest SA and its derivatives MeSa and acetylsalicylic acid (AAS) treatments induced a significant increase in total carotenoids in two plum cultivars at harvest and during storage [66].

## 4. Materials and Methods

### 4.1. Plant Material, Treatments and Experimental Design

The experiments were carried out on a commercial farm (Algro Farm, located in Palora, the province of Morona Santiago, Ecuador) of yellow pitahaya (*Selenicereus megalanthus* Haw.) plants grown under greenhouse conditions. The pitahaya plot consists in 4-year-old 1200 plants in an area of 2.5 ha (located at 1°41′00″ south latitude, 77°58′56.8″ west longitude, and altitude of 839 m.

For the experiments, 135 plants were chosen totally at random and divided in 3 blocks, and each block contained 3 plants in triplicate (n = 3) per treatment, as shown in Figure S1. In each plant, 3 fruits were marked to follow fruit growth (9 fruits per treatment) and the total number of fruits was 27.

Treatments were applied with a frequency of 15 days, starting at 57 days after full blossom (DAFB) and after 71, 86 and 102 DAFB, while the harvest was carried out at 126 DAFB. The elicitors were methyl salicylate (MeSa) and methyl jasmonate (MeJa) (both at 0.1 mM), while salicylic acid (SA) and oxalic acid (OA) were applied at 5 mM, based on previous experiments (Erazo-Lara et al., 2024) [29]. Treatments were performed by spraying 1.5 L per plant with freshly prepared solutions of MeSa, SA, MeJa and OA (purchased from Sigma-Aldrich, Madrid, Spain) containing 0.5% Tween-20 as the surfactant. Treatments were applied early in the morning and under favourable weather conditions (no rain or wind were forecasted). The control plants were treated with water + 0.5% Tween-20.

Pitahaya fruits were manually harvested at the commercial ripening stage based on fruit size, fruit weight (≈360 g), colour (light-green or yellow with green bracts) and the content of total soluble solids (TSS) with 15 °Brix [21]. In addition, the thorns were manually removed with a brush. For each treatment, 27 fruits (9 fruits per replicate) were picked and transferred by a refrigerated van to the airport and then shipped to Spain for the storage experiment. Another group of pitahayas (2 fruits per replicate and treatment) was used to determine TSS, the total acidity (TA) and fruit firmness at harvest (Day 0). The fruits were peeled to separate the pulp from the peel, for which the mineral content (macro- and micronutrients) and total phenolics were determined.

### 4.2. Postharvest Storage

Once the fruits arrived in Spain, they were stored in 10 °C and at a relative humidity of 85%. After 24, 38 and 52 days, one lot from each treatment (9 pitahayas) was taken from the cold room to analyse TSS, TA, firmness, total phenolic compounds, total carotenoids, and antioxidant activity from hydrophilic and lipophilic extracts.

### 4.3. Mineral Composition of the Peel

The peels corresponding to the fruits at harvest were submitted to dehydration in a heater at 65 °C until a constant weight was achieved. Moreover, 0.25 g of the peel samples from each treatment (in triplicate) was microwave-digested (CEM Mars One) after the addition of 10 mL of 1% nitric acid for 3 h and, afterwards, up to 50 mL with distilled water. Then, the aliquots of each sample were used to quantify the mineral concentration using the method of inductively coupled plasma mass spectrometry (ICP-MS) (Shimadzu icpms-2030, Kyoto, Japan). The mineral quantification was carried out by the use of standard curves of Ca, Mg, Na, P and K (macronutrients) and Fe, Mn, Cu and Zn (micronutrients), and the results were expressed as mg kg^−1^ dried weight.

### 4.4. Measurement of Quality Traits

Quality was evaluated in the pitahaya pulp obtained from the 9 fruits of each replicate by obtaining subsamples of the 3 fruits. TSS was determined as °Brix and expressed as g 100 g^−1^ with a Kem brand refractometer (RA-620 model). The percentage of TA (g 100 g^−1^) was determined by automatic titration (785 DMP Titrino, Metrohm, Herisau, Switzerland) by the use of 0.1 N NaOH up to pH 8.1. Fruit firmness (N mm^−1^) was evaluated with a texture analyser (Model TA.XTplus, Stable Micro Systems Ltd., Godalming, UK) by compression (3% of the pitahaya diameter), with the aid of a 75 mm flat plate probe and recording the force (N) and the distance (mm).

### 4.5. Bioactive Compounds and Antioxidant Activity

We used the methods described by Habibi et al. (2021) [67] and adapted them to the pitahaya fruit. The total phenolics were calculated by the Folin–Ciocalteau protocol by homogenizing 2 g of peel and pulp with 10 mL of extractant (20:80. water/methanol) and then submitted to centrifugation at 10,000× *g* at 4 °C for 20 min. The supernatant was added to the Folin–Ciocalteau reagent, with the results expressed as mg of gallic acid equivalent 100 g^−1^.

Total antioxidant activity was determined in both the aqueous solvent (hydrophilic) and organic solvent (lipophilic) by using the method of ABTS. Briefly, 5 g of pulp was added to 5 mL of phosphate buffer (pH = 6.8) and 10 mL of ethyl acetate, homogenizing for 2 min and then centrifuging at 10,000× *g* at 4 °C for 20 min. After the separation of the phases, each extract was measured in duplicate with an ABTS peroxidase system, and the results were expressed as mg 100 g^−1^ of Trolox equivalents. For total carotenoids, the organic phase was used and subjected to saponification with 10% KOH in MeOH solvent followed by extraction with diethyl ether and finally drying and dissolving in acetone. The total carotenoids were quantified by reading the absorbance at 450 nm in a spectrophotometer (UNICAM Helios-α spectrophotometer, Sci-Tek Instruments Ltd., Olney, UK) and expressed as μg β-carotene equivalent 100 g^−1^.

### 4.6. Statistical Analysis

All data in the paper were expressed as the mean ± standard error (SE) of three replicates. The data were subjected to one-way analysis of variance (ANOVA) for the variable treatment. A comparisons of the means were performed using a multiple range test (Tukey test) to find significant differences (*p* ≤ 0.05) among the treatments for each sampling date. All analyses were performed with the SPSS version 22 software package, and the SigmaPlot 11.0 software program was used for graphs.

## 5. Conclusions

It is clear that the postharvest storage of fruits is affected by preharvest factors, such as the ripening stage at harvest and quality traits (TSS, TA and firmness). The elicitors used in this study (MeSa, SA, JaMe and OA) induced a better quality of yellow pitahaya. Treated fruits with MeSa and SA had a higher TSS content and lower TA and firmness than those treated with MeJa and OA. The on-tree ripening process was affected by the type of elicitor since SA, JaMe and OA delayed the ripening process, while MeSa advanced the ripening. The peel of the yellow pitahaya is rich in minerals and bioactive compounds (polyphenols and carotenoids), and all of them showed a net enhancement with the use the elicitors. The macronutrients Ca, P, K and Mg were enhanced in MeSa- and OA-treated pitahayas. All micronutrients (Fe, Mn, Cu, Zn and Na) were higher in the MeSa-treated pitahaya. So far, peel has been considered as waste, but given the great amount of both nutritional and phytochemical compounds, the peel of yellow pitahaya would be a perfect candidate to be recycled and applied in the food industry. Since carotenoids and phenolics have been proven to have beneficial effects against degenerative diseases, preharvest treatments with these elicitors would provide healthier yellow pitahayas for human consumption. The preharvest spray is recommended as the optimal method due to its fast absorption and high effectiveness as an alternative to postharvest treatments. The preharvest elicitors demonstrated an improvement of yellow pitahaya quality during postharvest storage quality through maintaining fruit firmness, TSS and TA. Tropical fruits are very sensitive to develop chilling injury (CI) when stored at sub-optimal temperatures. For that reason, we stored the fruits at 10 °C (non-CI temperature), but in future, the use of these elicitors and the possibility to use lower temperatures for storing yellow pitahaya deserves further investigation.

## Data Availability

The original contributions generated for this study are included in the article; the data presented in this study are available on request from the authors.

## Supplementary Materials

The following supporting information can be downloaded at: https://www.mdpi.com/article/10.3390/ijms25105435/s1.
