# Supplementary material for: Preharvest Elicitors as a Tool to Enhance Bioactive Compounds and Quality of Both Peel and Pulp of Yellow Pitahaya (Selenicereus megalanthus Haw.) at Harvest and during Postharvest Storage
Source: Int J Mol Sci. 2024 May 16;25(10):5435. doi: 10.3390/ijms25105435 (PMC11121277; doi:10.3390/ijms25105435)

Figure S1. Distribution of the pitahaya plants in 3 blocks at random for the elicitor applications.

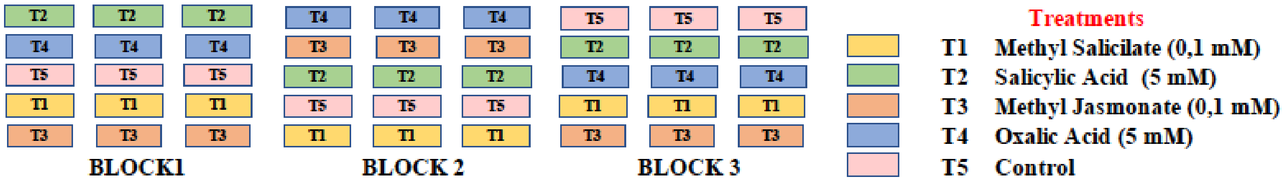

Supplement: Supplementary file 1 [file ijms-25-05435-s001.zip › ijms-2988504-supplementary.pdf]
